# Lidocaine Promoted Ferroptosis by Targeting miR-382-5p /SLC7A11 Axis in Ovarian and Breast Cancer

**DOI:** 10.3389/fphar.2021.681223

**Published:** 2021-05-26

**Authors:** Dan Sun, Ying-Chun Li, Xiao-Yu Zhang

**Affiliations:** ^1^ Second Gynecology Department, Cangzhou Central Hospital, Cangzhou, China; ^2^ Department of Thyroid and Breast Ⅲ, Cangzhou Central Hospital, Cangzhou, China

**Keywords:** ovarian cancer, breast cancer, lidocaine, ferroptosis, miR-382-5p, SLC7A11

## Abstract

Ovarian and breast cancer are prevalent female malignancies with increasing occurrence incidence and metastasis, significantly affecting the health and life quality of women globally. Anesthetic lidocaine has presented anti-tumor activities in the experimental conditions. However, the effect of lidocaine on ovarian and breast cancer remains elusive. We identified the important function of lidocaine in enhancing ferroptosis and repressing progression of ovarian and breast cancer. Our data showed that lidocaine further repressed erastin-inhibited ovarian and breast cancer cell viabilities. The treatment of lidocaine induced accumulation of Fe^2+^, iron and lipid reactive oxygen species (ROS) in ovarian and breast cancer cells. The ovarian and breast cancer cell proliferation was suppressed while cell apoptosis was induced by lidocaine *in vitro*. Lidocaine attenuated invasion and migration of ovarian and breast cancer cells as well. Regarding the mechanism, we found that lidocaine downregulated solute carrier family 7 member 11 (SLC7A11) expression by enhancing microRNA-382-5p (miR-382-5p) in the cells. The inhibition of miR-382-5p blocked lidocaine-induced ferroptosis of ovarian and breast cancer cells. MiR-382-5p/SLC7A11 axis was involved in lidocaine-mediated inhibition of ovarian and breast cancer cell proliferation *in vitro*. The miR-382-5p expression was down-regulated but SLC7A11 expression was up-regulated in clinical ovarian and breast cancer samples. Furthermore, the treatment of lidocaine repressed tumor growth of ovarian cancer cells *in vivo*, in which the miR-382-5p expression was increased while SLC7A11 expression was decreased. Consequently, we concluded that the lidocaine promoted ferroptosis by miR-382-5p/SLC7A11 axis in ovarian and breast cancer cells. The clinical value of lidocaine in the treatment of ovarian and breast cancer deserves to be proved in detail.

## Introduction

Breast cancer and ovarian cancer are the most frequently occurred gynecological cancers globally, severely threatened the life quality of females (de Sousa Cunha et al., 2019; Siegel et al., 2020). Breast cancer has gain great attentions caused by its constantly increasing incidence despite of the improvement in treatment (Britt et al., 2020). Moreover, ovarian cancer represents a major clinical challenge due to the frequent late diagnosis at advanced tumor stage (Jayson et al., 2014). Ferroptosis is a novel form of cell death related to iron-caused accumulation of lipid reactive oxygen species (ROS) (Dixon et al., 2012). Accumulating studies have indicated that targeting ferroptosis may be a feasible strategy for therapy of breast cancer and ovarian cancer (Li et al., 2020). It was proposed that triple negative breast cancer (TNBC) patients were more sensitive to ferroptosis than estrogen receptor (Wang et al., 2021)-positive patients, which provided a potential therapeutic strategy for TNBC patients (Doll et al., 2017). Ferroptosis also mediated platinum tolerance in ovarian cancer (Wang et al., 2021).

Lidocaine is a local anesthetic drug derived from cocaine, and widely used in clinical application such as anti-bacteria, anti-inflammation, local skin anesthesia and so on (Caracas et al., 2009; Adler et al., 2017; Cizmarikova et al., 2020). An increasing number of evidences have demonstrated that lidocaine could affect the development of multiple tumors including ovarian and breast cancer (Gao et al., 2018; Khan et al., 2019; Liu et al., 2021). For example, it was reported that intravenous infusion of lidocaine during operation was associated with improved overall survival of patients with pancreatic cancer (Zhang et al., 2020). Gao and colleagues suggested that the administration of lidocaine could enhance the apoptosis of breast cancer cells caused by cisplatin, as well as alleviating their metastasis *in vivo* (Gao et al., 2018). Moreover, lidocaine could also suppress the growth and metastasis of ovarian cancer (Liu et al., 2021). However, whether ferroptosis is involved in the function of lidocaine in breast and ovarian cancer is still unknown.

MicroRNA (miRNA) is a class of endogenous short-sequenced non-coding RNAs (Lee and Dutta, 2009). MiRNAs commonly function *via* interacting with the mRNA of targeted genes and impede their translation to functional proteins, which could lead to the regulation of various biological processes during carcinogenesis, such as growth, angiogenesis and metastasis (Lee and Dutta, 2009). For instance, miR-382-5p acted as a competitive endogenous RNAs (ceRNA) of circRNA-UBAP2 to promote proliferation and suppress apoptosis of ovarian cancer cells (Xu et al., 2020). Recent studies even revealed the involvement of miRNA in lidocaine-related suppression of cancer (Sui et al., 2019; Sun and Sun, 2019). Lidocaine regulated the expression of EGFR through miR-539, which further hindered the proliferation and metastasis of lung cancer cell (Sun and Sun, 2019). However, the detailed involvement of miR-382-5p in lidocaine-affected progression of ovarian cancer and breast cancer is not clear. SLC7A11 is the gate keeper for antiporter of cystine-glutamate, also involved in cancer development (Liu et al., 2019), Newest study have indicated that SLC7A11 mediated the radiotherapy and immunotherapy of cancers *via* ferroptosis, which implied the role of SLC7A11 in regulating ferroptosis of cancers (Lang et al., 2019).

In this work, we disclosed the inhibitory effect of lidocaine on growth and metastasis of breast cancer and ovarian cancer cells *via* affecting ferroptosis process. Mechanistically, lidocaine administration stimulated ferroptosis through upregulating miR-382-5p and the subsequent suppression of SLC7A11 level. Our work suggested a miR-382-5p/SLC7A11 regulatory axis mediating lidocaine-induced ferroptosis in breast and ovarian cancer, provided new basis for therapy of female malignancies.

## Materials and Methods

### Cell Lines and Transfection

Ovarian cancer cell line SKOV-3 and breast cancer cell line T47D were purchased from the Shanghai Institute of Life Sciences at the Chinese Academy of Sciences (Shanghai, China), and cultured in the DMEM cell culturing medium (Hyclone, United States) containing 10% FBS (Gibco, United States) in a 37°C humidified atmosphere filled with 5% CO_2_.

The miR-382-5p mimics, miR-382-5p inhibitor, siSLC7A11, and the scramble controls (NC) were designed and purchased from RioBio (China). SKOV-3 and T47D cells were plated in 6-well plates and incubated 12 h before transfection. Next, miRNA or siRNA was mixed with lipofectamine 2,000 in the opti-MEM medium and added in each well. After a 24-hour transfection, cells were collected to perform subsequent experiments.

### Patients and Tissue Samples

We included 38 ovarian cancer patients and 50 breast cancer patients, who received surgical operation in Cangzhou Central Hospital. Tumor tissues were collected during operation and subjected to real-time PCR to evaluate the levels of SLC7A11 and miR-382-5p. All experiments have acquired the consents of patients and were performed under approval of Cangzhou Central Hospital.

### MTT

SKOV-3 and T47D cells were plated in 96-well plates at a density of 5 × 10^3^ cell/well, and cultured for 12 h to form a monolayer. Subsequently, lidocaine (Sigma, United States) diluted by cell culture medium was added into each well and incubated for indicated time (24, 48, and 72 h). At the end time point, 10 μL MTT (5 mg/ml) were added into each well and the cells were cultured for another 4 h. Then the medium was discarded and 150 μL DMSO was added into each well to incubate for 10 min in dark. The absorbance values were detected at 490 nm.

### Colony Formation

SKOV-3 and T47D cells were digested and suspended in DMEM as single cell. A total number of 1,000 cells were seeded into each well of 6-well plate, followed by administration of lidocaine 12 the next day. The medium was replaced with fresh DMEM containing lidocaine every three days. The cells were cultured for 15 days until visible clones formed. Subsequently, the clones were fixed, stained by violate crystal (Sigma) for 30 min at room temperature and captured in a microscope (Olympus, Japan).

### Cell Apoptosis

The apoptotic cells were detected by an Apoptosis Assay Kit (Thermo, United States) under manufacturer’s protocol. Briefly, SKOV-3, and T47D cells treated with lidocaine were collected, washed and double stained by FITC-Annexin V and PI reagents. The A flow cytometer (BD Biosciences, United States) was adopted to measure the cells undergoing early and late apoptosis.

### Transwell

The transwell chambers (Corning, United States) covered with or without Matrigel (Corning) were used to check the invasion or migration ability of SKOV-3 and T47D cells. Cells were placed in the upper chamber with DMEM medium containing no FBS, while the lower chambers were filled with complete culturing medium with 10% FBS. After incubation for 48 h, the upper chambers were washed with PBS and stained with violate crystal.

### Wound Healing

Wound healing assay was performed to determine cell migration. SKOV-3 and T47D cells were placed in 6-well plates at a density of 1 × 10^6^ cell per well and cultured for 12 h to form a monolayer confluence. Then a 200 μL pipet was used to gently scratch a line on the monolayer. Then the medium was replaced by fresh FBS-free medium containing lidocaine. The pictures of scratches were taken at 0, 6, and 12 h.

### Detection of Iron and ROS Level

Iron assay kit (Beyotime) and BODIPY C-11 (Sigma) staining were conducted to measure the intracellular Fe^2+^ level and the lipid ROS level in SKOV-3 and T47D cells under the manufacturers’ instructions, separately.

### Western Blotting

The proteins from SKOV-3 and T47D cells or tumor tissues were extracted by ice-cold RIPA lysis buffer (Solarbio, China) added with protease inhibitor cocktail (Thermo), and separated by 8–10% SDS-PAGE. The separated proteins were transferred to NC membranes. The blots were blocked and incubated with specific primary antibodies against GPX4 (1:1000, Proteintech, China), SLC7A11 (1:1000, Proteintech), Bcl-2 (1:1000, abcam, United States), Bax (1:1000, abcam, United States), caspase3 (1:1000, abcam, United States), cleaved-caspase3 (1:1000, abcam, United States), RIPK3 (1:1000, abcam, United States), and β-actin (1:1000, Proteintech) overnight at 4°C. Following washing with PBST, the blots were incubated at room temperature by peroxidase-conjugated secondary antibodies (1:1000, Proteintech). Proteins were visualized by using an ECL reagent (Millipore, United States) in an Image Lab Software (BIO RAD, United States).

### RNA Quantification

Total RNA from tumor tissues and SKOV-3 and T47D cells were isolated using TRIzol® reagent (Sigma, United States). Quantitative real-time PCR (qPCR) for miR-382-5p and SLC7A11 mRNA was performed using a SYBR Green Mix (Thermo) and measured by a Bio-Rad CFX96 real-time PCR system (United States). Relative gene expression levels were calculated using a 2^−ΔΔCt^ method. The normalization was achieved by using U6 and GAPDH as the internal control of miR-382-5p and SLC7A11 mRNA. The primers are as follows: SLC7A11 sense: 5'- TCT​CCA​AAG​GAG​GTT​ACC​TGC -3' and anti-sense: 5'- AGA​CTC​CCC​TCA​GTA​AAG​TGA​C -3'; miR-382-5p sense: 5'-ACA​CTC​CAG​CTG​GGA​AAG​TGC​TTC​CC-3' and anti-sense: 5'-CTC​AAC​TGG​TGT​CGT​GGA-3'; GAPDH sense: 5'-TGG​GTG​TGA​ACC​ACG​AGA​A-3' and anti-sense: 5'-GGC​ATG​GAC​TGT​GGT​CAT​GA-3'; U6, sense 5'-GCTTCGGCAGCA CATATAATAAAAT-3'; anti-sense 5'-CGC​TTC​ACG​AAT​TTG​CGT​GTC​AT-3'.

### Luciferase Reporter Gene

The predicted interaction and binding site between miR-382-5p and SLC7A11 was obtained by TargetScan website (http://www.targetscan.org/vert_72/). To clarify the interaction between miR-382-5p and SLC7A11, the luciferase reporter plasmids psiCHECK-2 (Promega, United States) inserted with wide type (WT) or mutated (Mut) 3'UTR of SLC7A11 were constructed. The T47D and SKOV-3 cells were transfected with SLC7A11-WT or SLC7A11-Mut together with miR-382-5p mimics or NC and pRL-TK as the internal control. The luciferase activity was measured in a microplate reader (PerkinElmer) by using a dual-luciferase assay system (Promega) under the manufacturer’s instruction.

### Xenograft Assays

All animal experiments were performed under the approvement of Animal Ethics Committee of the Cangzhou Central Hospital. SPF-level male nude mice aged 5–6 weeks and weighted around 20 g were purchased from Vitalriver (China). All mice were maintained in a 12-hour circadian rhythm, and had free access to water and food. Cancer cells were subcutaneously injected into the right flank of mice. Lidocaine was administrated to mice at a dose of 1.5 mg per kg injected through the vail tails. For control group, the mice were treated with saline. Tumor volume and mice body weight were monitored every 5 days. The tumor size was calculated *via* the following formula: length × width^2^/2.

### Statistics

Each experiment was performed at least three times. Date was shown as mean ± standard deviation (SD), and analyzed by SPSS software (version 19.0). The statistical comparison between groups were conducted *via* Student’s t-tests or one-way analyses of variance (ANOVA). *p* < 0.05 was considered as statistically significant.

## Results

### Lidocaine Induces Ferroptosis of Ovarian and Breast Cancer Cells

**FIGURE 1:**
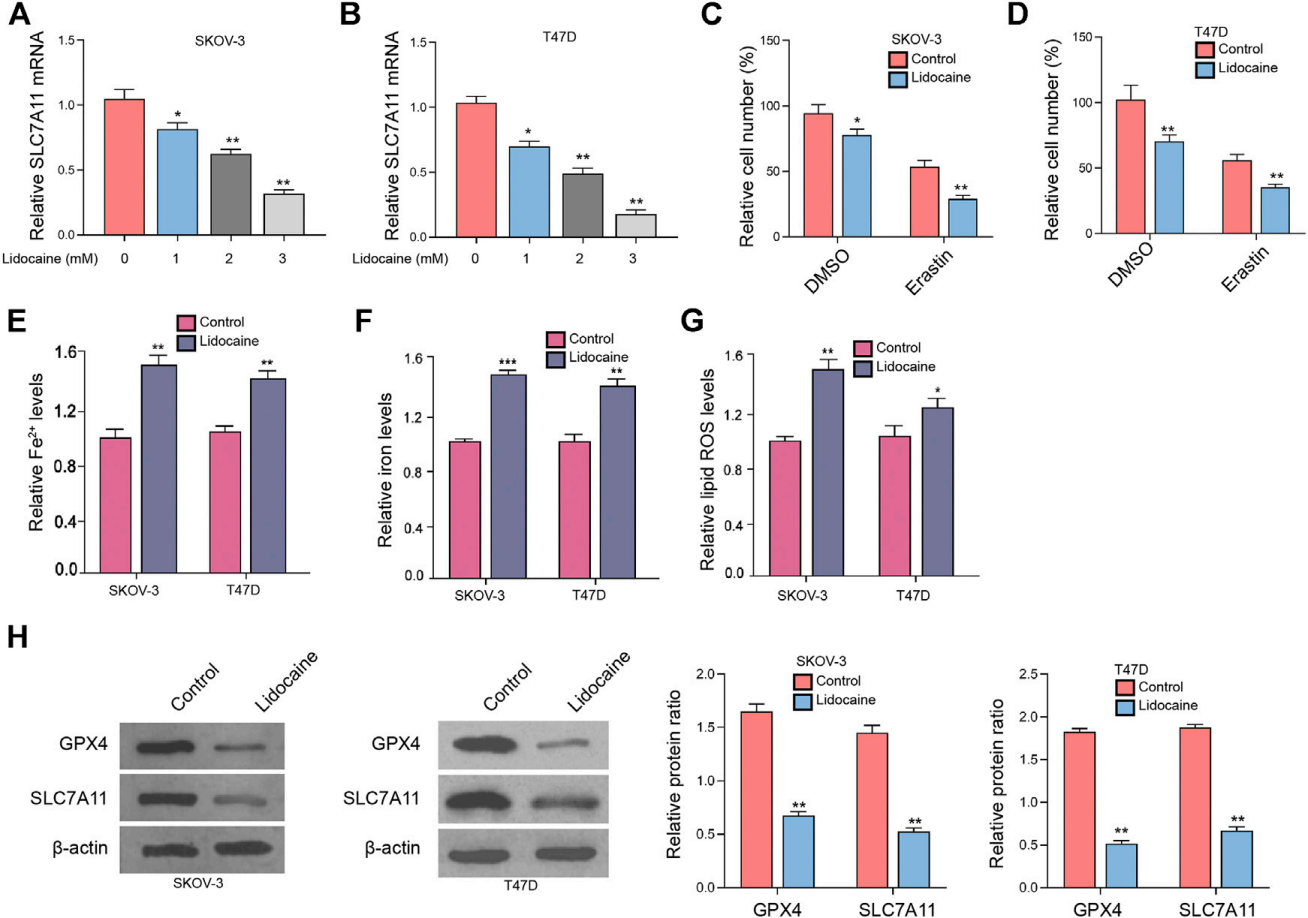
Lidocaine induces ferroptosis of ovarian and breast cancer cells. **(A,B)** SKOV-3 and T47D cells were treated with lidocaine at the indicated concentrations. The mRNA expression of SLC7A11 was analyzed by qPCR. **(C,D)** SKOV-3 and T47D cells were co-treated with erastin (5 mmol/L) and lidocaine (3 mM). The cell viability was detected by MTT assays after 48 h of the treatment. **(E–H)** SKOV-3 and T47D cells were treated with lidocaine (3 mM). The Fe^2+^ **(E)**, iron (Tesfay et al., 2019), and lipid ROS levels **(G)** were detected. **(H)** The expression of GPX4 and SLC7A11 was measured by Western blot analysis. The results were quantified using ImageJ software. mean ± SD, **p < 0.05, **p < 0.01. The experiments were performed independently three times.

We were interested in the function of lidocaine in regulating ferroptosis of ovarian and breast cancer cells. Our data showed that lidocaine inhibited SLC7A11 mRNA expression in a dose-dependent manner, in which 3 mM lidocaine presented the highest effect and was selected in the subsequent analysis (Figures 1A,B). The co-treatment of lidocaine and erastin was able to enhance effect of erastin on the inhibition of SKOV-3 and T47D cells (Figures 1C,D). Meanwhile, total iron (iron) and ferrous iron (Fe^2+^) were analyzed in the cells. The levels of Fe^2+^ and iron were induced by lidocaine in SKOV-3 and T47D cells (Figures 1E,F). In addition, lidocaine promoted lipid ROS accumulation in SKOV-3 and T47D cells (Figure 1G). The expression of SLC7A11 and GPX4 was repressed by lidocaine in SKOV-3 and T47D cells (Figure 1H). Taken together, these data suggest that lidocaine induces ferroptosis of ovarian and breast cancer cells.

### Lidocaine Reduces the Proliferation of Ovarian and Breast Cancer Cells *In Vitro*

**FIGURE 2:**
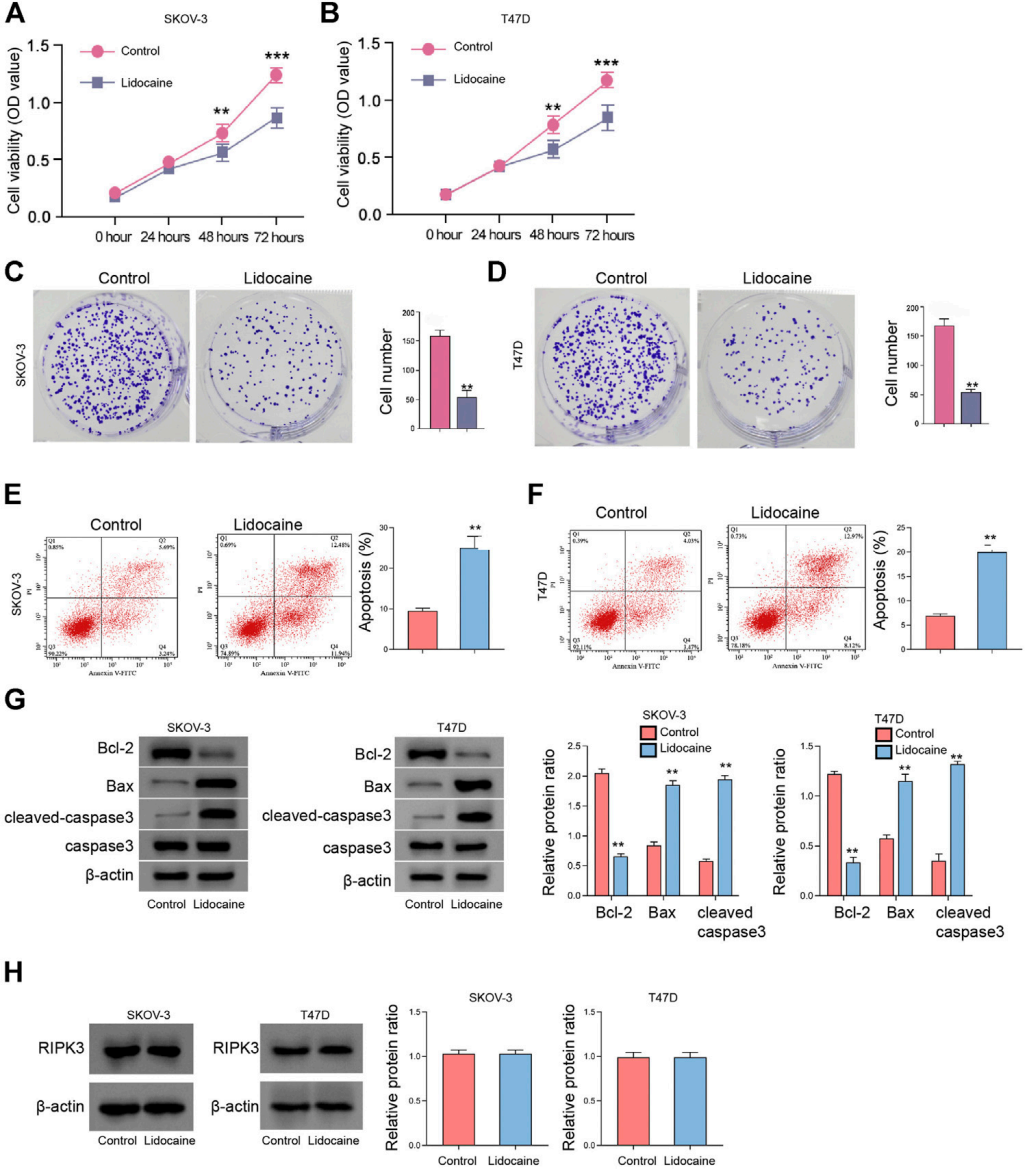
Lidocaine reduces the proliferation of ovarian and breast cancer cells *in vitro*. **(A–H)** SKOV-3 and T47D cells were treated with lidocaine (3 mM). **(A**,**B)** The cell viability was detected by MTT assays. **(C,D)** The cell proliferation was measured by colony formation assays. **(E,F)** The apoptosis was analyzed by flow cytometry. **(G)** The expression of Bcl-2, Bax, caspase3, and cleaved-caspase3 was detected by Western blot. The results were quantified using ImageJ software. **(H)** The expression of RIPK3 was measured by Western blot. The results were quantified using ImageJ software. mean ± SD, **p < 0.01. The experiments were performed independently three times.

We then observed that the SKOV-3 and T47D cell viabilities were reduced by lidocaine (Figures 2A,B). Consistently, the colony formation numbers of SKOV-3 and T47D cells were suppressed by the treatment of lidocaine (Figures 2C,D). The apoptosis of SKOV-3 and T47D cells was induced by lidocaine (Figures 2E,F), indicating that lidocaine reduces the proliferation of ovarian and breast cancer cells *in vitro.* Moreover, the expression of apoptosis and necroptosis markers was detected. We found that Bcl-2 expression was repressed and Bax and cleaved-caspase3 expression was induced by lidocaine in SKOV-3 and T47D cells (Figure 2G). Meanwhile, the treatment of lidocaine failed to affect necroptosis marker RIPK3 expression in the cells (Figure 2H). Together these results indicate that lidocaine reduces the proliferation of ovarian and breast cancer cells *in vitro.*

### Lidocaine Suppresses Invasion and Migration of Ovarian and Breast Cancer Cells *In Vitro*

**FIGURE 3:**
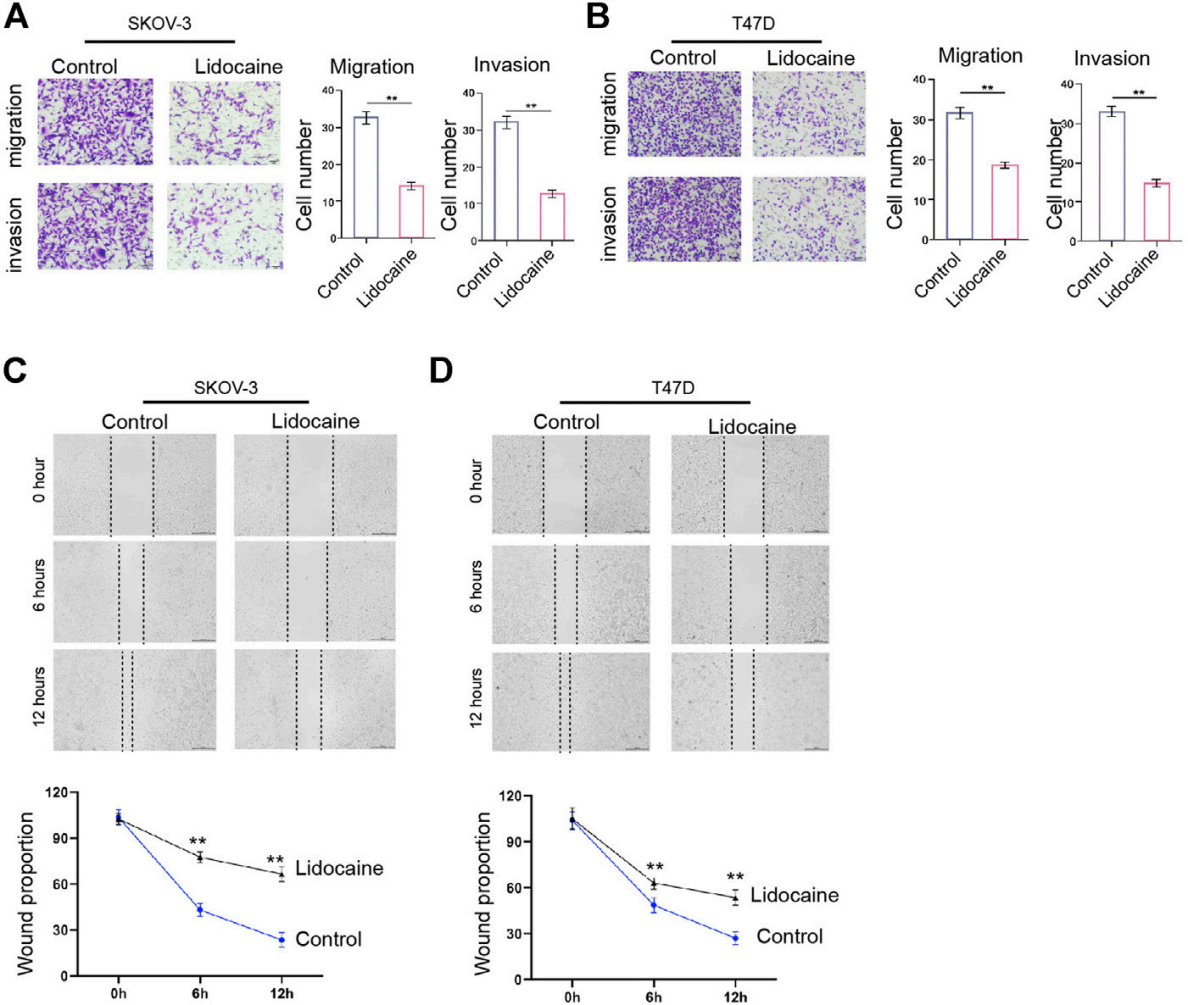
Lidocaine suppresses invasion and migration of ovarian and breast cancer cells *in vitro*. **(A–D)** SKOV-3 and T47D cells were treated with lidocaine (3 mM). **(A,B)** The cell invasion and migration were measured by transwell analysis. **(C,D)** The cell migration was analyzed by wound healing analysis. mean ± SD, **p < 0.01. The experiments were performed independently three times.

Next, we also found that the treatment of lidocaine attenuated invasion and migration of SKOV-3 and T47D cells (Figures 3A,B). Similarly, the wound healing abilities of SKOV-3 and T47D cells were repressed by the treatment of lidocaine *in vitro* (Figures 3C,D). Together these results imply that lidocaine suppresses invasion and migration of ovarian and breast cancer cells *in vitro*.

### Lidocaine Inhibits SLC7A11 Expression by Upregulating miR-382-5p

**FIGURE 4:**
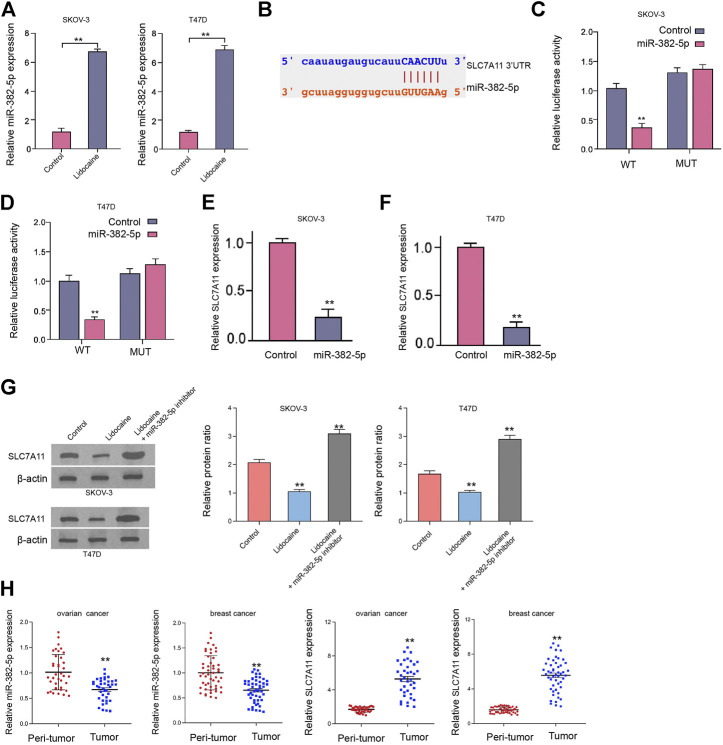
Lidocaine inhibits SLC7A11 expression by upregulating miR-382-5p. **(A,B)** SKOV-3 and T47D cells were treated with lidocaine (3 mM). The expression of miR-382-5p was analyzed by qPCR assays. **(C–F)** SKOV-3 and T47D cells were treated with miR-382-5p mimic. **(C,D)** The luciferase activity of SLC7A11 mRNA 3’UTR was analyzed. **(E,F)** The mRNA levels of SLC7A11 were examined by qPCR. **(G)** The protein expression of SLC7A11 was detected by Western blot analysis in SKOV-3 and T47D cells co-treated with lidocaine and miR-382-5p inhibitor. **(H)** The expression of miR-382-5p and SLC7A11 was detected by qPCR in clinical ovarian cancer tissues (*n* = 38) and breast cancer tissues (*n* = 50) and the related adjacent tissues. mean ± SD, **p < 0.01. The experiments were performed independently three times.

Next, our data demonstrated that the expression of miR-382-5p was up-regulated by lidocaine in SKOV-3 and T47D cells (Figure 4A). We predicted the binding site of miR-382-5p and SLC7A11 in a bioinformatic analysis (Figure 4B). The mRNA expression along with the luciferase activity of SLC7A11 were inhibited by miR-382-5p mimic in SKOV-3 and T47D cells (Figures 4C–F). The protein levels of SLC7A11 were repressed by the treatment of lidocaine while the inhibition of miR-382-5p blocked the effect of lidocaine in SKOV-3 and T47D cells (Figure 4G). The miR-382-5p expression was down-regulated but SLC7A11 expression was up-regulated in clinical ovarian cancer tissues (n = 38) and breast cancer (n = 50) tissues compared with the adjacent tissues (Figure 4H). Taken together, these data suggest that lidocaine inhibits SLC7A11 expression by upregulating miR-382-5p.

Moreover, we identified that the SKOV-3 and T47D cell viabilities were reduced by miR-382-5p (Supplementary Figures S1A,B). The colony formation numbers of SKOV-3 and T47D cells were suppressed by the treatment of miR-382-5p mimic (Supplementary Figures S1C,D). The apoptosis of SKOV-3 and T47D cells was induced by miR-382-5p (Supplementary Figures S1E,F)*.* The levels of Fe^2+^, iron, lipid ROS were induced by miR-382-5p in SKOV-3 and T47D cells (Supplementary Figures S1G–I)*.* Together these results indicate that miR-382-5p represses proliferation and induces ferroptosis of ovarian and breast cancer cells *in vitro.*

### The Inhibition of miR-382-5p Blocks Lidocaine-Induced Ferroptosis of Ovarian and Breast Cancer Cells

**FIGURE 5:**
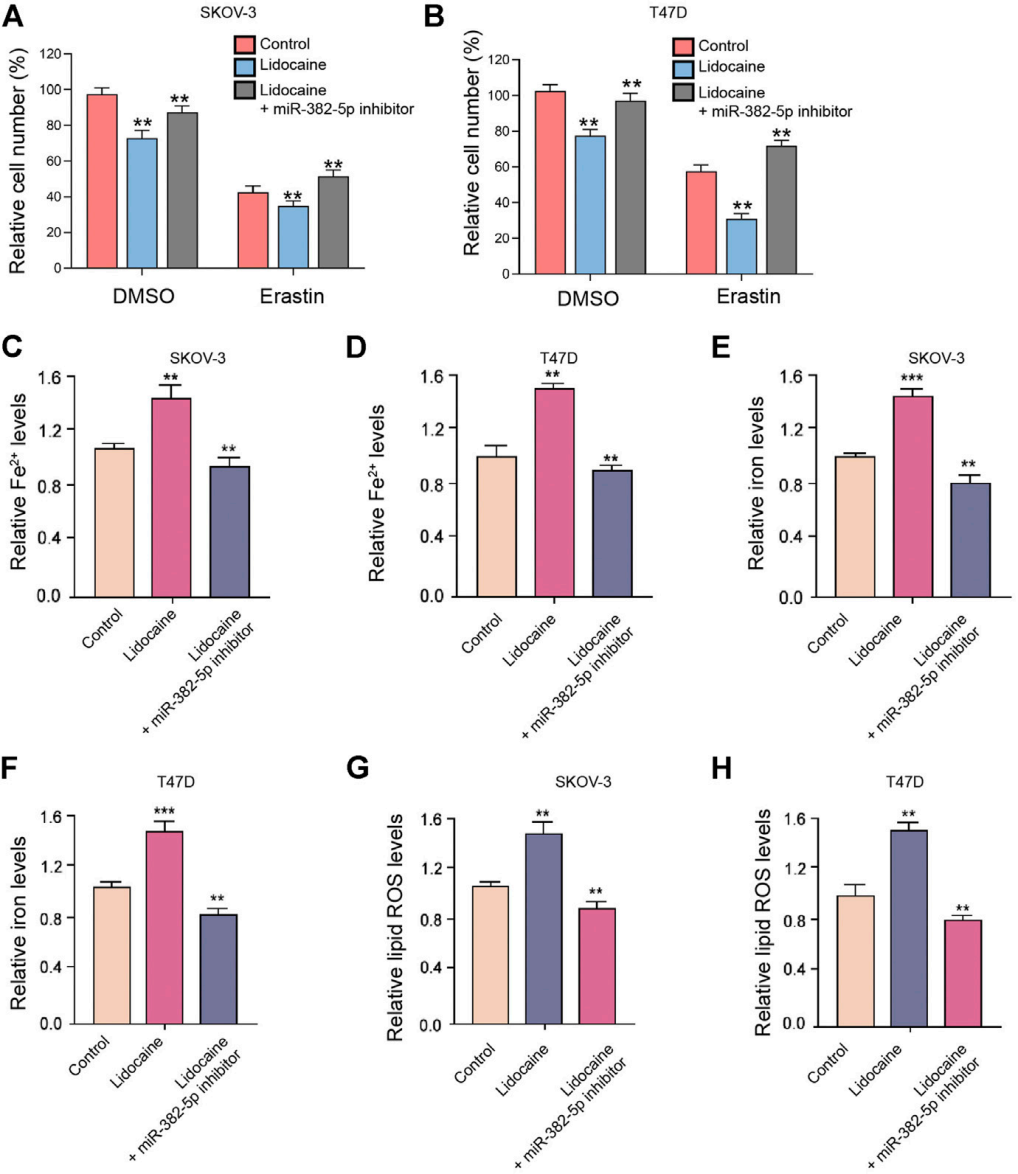
The inhibition of miR-382-5p blocks lidocaine-induced ferroptosis of ovarian and breast cancer cells. **(A,B)** The erastin (5 mmol/L) -stimulated SKOV-3 and T47D cells were co-treated with miR-382-5p inhibitor and lidocaine (3 mM). The cell viability was detected by MTT assays after 48 h of the treatment. **(C–H)** SKOV-3 and T47D cells were co-treated with miR-382-5p inhibitor and lidocaine. The Fe^2+^ **(C,D)**, iron **(E,F)**, and lipid ROS levels **(G,H)** were detected. mean ± SD, **p < 0.01. The experiments were performed independently three times.

Next, we found that miR-382-5p inhibition rescued cell viabilities repressed by lidocaine in erastin-stimulated SKOV-3 and T47D cells (Figures 5A,B). Moreover, the levels of Fe^2+^, iron, and lipid ROS were enhanced by the treatment of lidocaine, in which the miR-382-5p inhibitor reversed these levels in SKOV-3 and T47D cells (Figures 5C–H).

**FIGURE 6:**
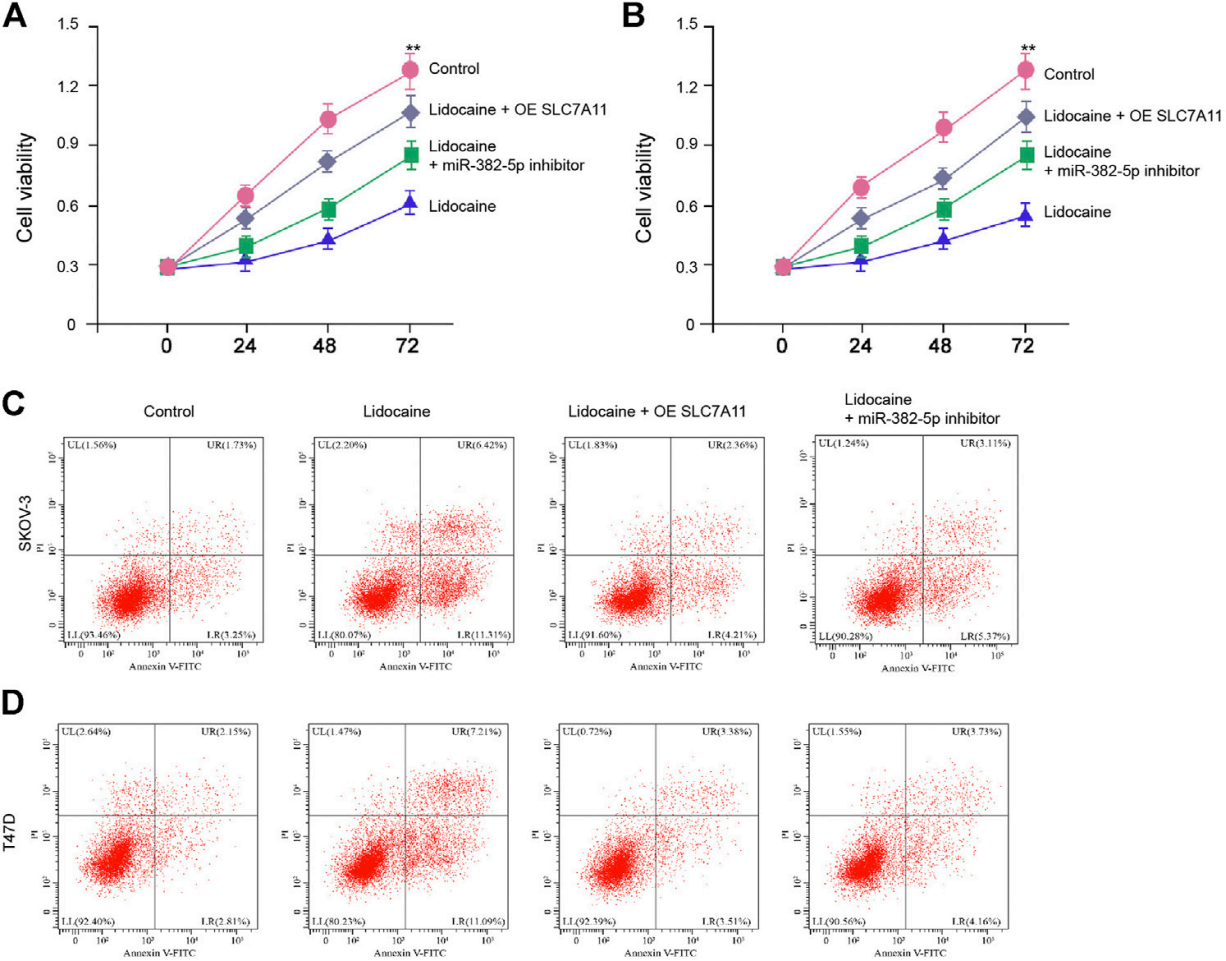
Lidocaine/miR-382-5p axis reduces the proliferation of ovarian and breast cancer cells by targeting SLC7A11 *in vitro*. **(A–D)** SKOV-3 and T47D cells were co-treated with lidocaine (3 mM) and miR-382-5p inhibitor or SLC7A11 reconstitution vectors. **(A,B)** The cell viability was detected by MTT assays. **(C,D)** The apoptosis was analyzed by flow cytometry. mean ± SD, **p < 0.01. The experiments were performed independently three times.

We then showed that the treatment of lidocaine inhibited cell proliferation and stimulated cell apoptosis of SKOV-3 and T47D cells while miR-382-5p inhibitor or SLC7A11 overexpression was able to reverse the effect of lidocaine on SKOV-3 and T47D cell proliferation and apoptosis *in vitro* (Figures 6A–D). Taken together, these data suggest that inhibition of miR-382-5p blocks lidocaine-induced ferroptosis of ovarian and breast cancer cells.

### Lidocaine Attenuates Proliferation of Ovarian Cancer Cells *In Vivo*

**FIGURE 7:**
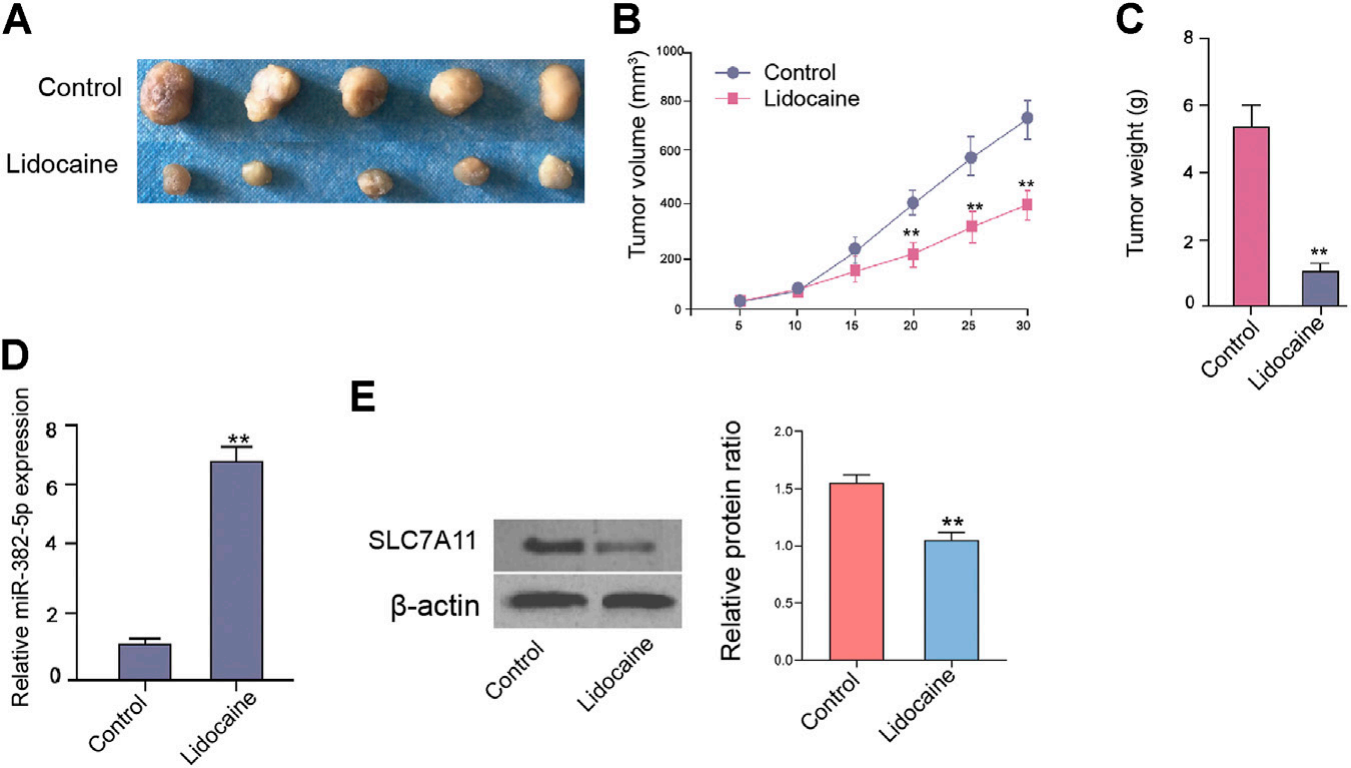
Lidocaine attenuates proliferation of ovarian cancer cells *in vivo*. The nude mice were injected with SKOV-3 cells and treated with lidocaine (1.5 mg/kg). The tumor tissues **(A)**, tumor volume **(B)**, and tumor weight **(C)** were shown. **(D)** The expression of miR-382-5p was analyzed by qPCR assays. **(E)** The protein expression of SLC7A11 was detected by Western blot analysis. The results were quantified using ImageJ software. N = 5, mean ± SD, **p < 0.01.

The tumorigenicity analysis further demonstrated that the tumor growth of SKOV-3 cells was attenuated by the treatment of lidocaine in the nude mice (Figures 7A–C). As expected, the expression of miR-382-5p was enhanced and SLC7A11 expression was reduced in the tumor tissues of lidocaine-treated mice compared with that in control group (Figures 7D,E). Together these results indicate that lidocaine attenuates proliferation of ovarian cancer cells *in vivo.*

## Discussion

Ovarian and breast cancer are severe and common female malignancies with high recurrence and metastasis rates. Despite the anesthetic lidocaine has been identified to present potential anti-tumor effects, the functions of lidocaine in ovarian and breast cancer are unclear. In the present study, we reported a critical role of lidocaine in modulating ferroptosis of ovarian and breast cancer cells.

Multiple local anesthetics, such as bupivacaine and levobupivacaine, have presented the inhibitory effect on cancer development (Xuan et al., 2016; Li et al., 2019). The Regional anesthesia potentially benefits the clinical outcomes of cancer patients (Xuan et al., 2015; Wall et al., 2019). Lidocaine has demonstrated significant anti-cancer activities in the previous investigations. It has been reported that lidocaine represses cytotoxicity resistance by down-regulating miR-21 in DDP-resistant lung cancer cells (Yang et al., 2019). Lidocaine enhances apoptosis and reduces proliferation by inducing miR-520a-3p expression to inhibit EGFR in colorectal cancer cells (Qu et al., 2018). Lidocaine enhances apoptosis and reduces proliferation of cervical cancer cells by targeting the lncRNA MEG3/miR-421/BTG1 axis (Zhu and Han, 2019). Our data further found that lidocaine induced an inhibitory effect on ovarian and breast cancer cell proliferation *in vitro* and *in vivo*. It identifies an unreported function of lidocaine in attenuating the progression of female cancers, including ovarian and breast cancer. The clinical application of lidocaine in the treatment of ovarian and breast cancer are needed to prove in detail. Ferroptosis, as an emerging programmed cell death, plays critical functions in both of ovarian and breast cancer. These reports indicate that targeting ferroptosis may be the potential anti-tumor therapies in ovarian and breast cancer. Frizzled-7 regulates platinum-resistant ovarian cancer cells by modulating ferroptosis (Wang et al., 2021). Stearoyl-CoA desaturase 1 reduces ferroptosis of ovarian cancer cells (Tesfay et al., 2019). Ferroptosis is enhanced by the treatment of lapatinib and siramesine in breast cancer (Ma et al., 2016). The PI3K/AKT/mTOR signaling inhibits ferroptosis by SREBP-regulated lipogenesis in breast cancer (Yi et al., 2020). In this study, we found that lidocaine induced ferroptosis of both ovarian and breast cancer cells. It suggests that lidocaine may inhibit malignant progression of ovarian and breast cancer by stimulating ferroptosis, providing the valuable evidence of the relationship of lidocaine and ferroptosis. Moreover, there are some limitations of this study. For example, more direct evidence about the effect of lidocaine on ferroptosis and apoptosis need to explore in future investigations. In addition, the function of lidocaine in the modulation of ferroptosis and apoptosis should be validated in mouse model. Meanwhile, circRNA-UBAP2 represses apoptosis and contributes to the proliferation of ovarian cancer by miR-382-5p/PRPF8 axis (Xu et al., 2020). SNHG1 promotes invasion and proliferation of breast cancer by targeting miR-382 (Zheng et al., 2019). Furthermore, it has been identified that miR-382-5p can induces apoptosis by targeting PRPF8 and SPIN1 in ovarian cancer and lung cancer, respectively (Chen et al., 2020; Xu et al., 2020). Our data showed that lidocaine up-regulated the expression of miR-382-5p to reduce SLC7A11 expression. The inhibition of miR-382-5p blocked lidocaine-mediated ferroptosis in ovarian and breast cancer cells. Our finding indicates a new correlation of miR-382-5p with lidocaine in the regulation of ferroptosis during cancer development. MiR-382-5p/SLC7A11 axis may be just one of the downstream mechanisms underlying lidocaine-repressed cancer progression and more mechanisms are needed to be explored in future investigation.

Consequently, we concluded that the lidocaine promoted ferroptosis by miR-382-5p/SLC7A11 axis in ovarian and breast cancer cells. The clinical value of lidocaine in the treatment of ovarian and breast cancer deserves to be proved in detail.

## Data Availability

The original contributions presented in the study are included in the article/Supplementary Material, further inquiries can be directed to the corresponding author.
