# Supplementary figures and images for: Lidocaine Promoted Ferroptosis by Targeting miR-382-5p /SLC7A11 Axis in Ovarian and Breast Cancer
Source: Front Pharmacol. 2021 May 26;12:681223. doi: 10.3389/fphar.2021.681223 (PMC8188239; doi:10.3389/fphar.2021.681223)

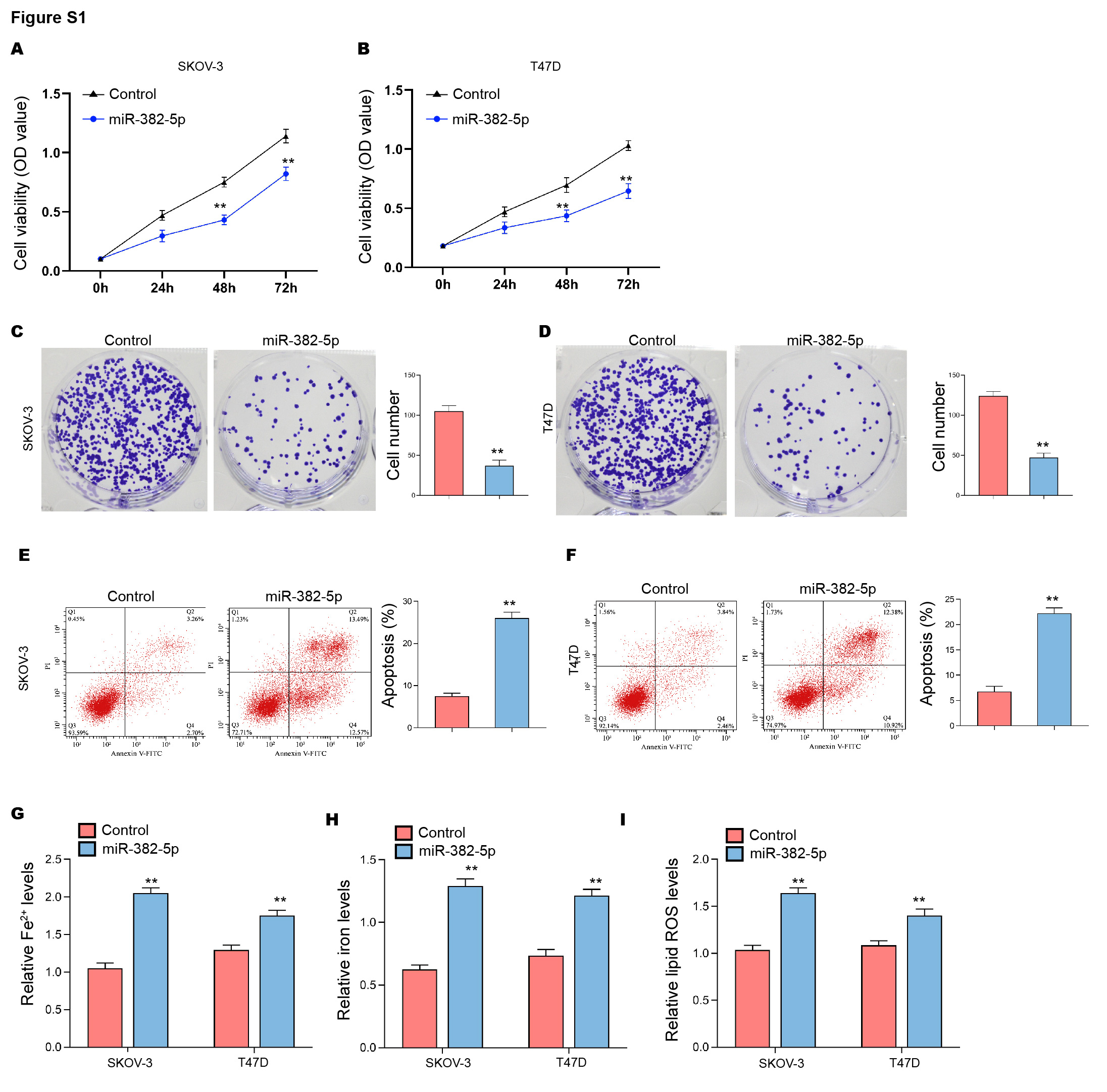

Supplement: Supplementary file 1 [file Image1.JPEG]
